# Supplementary material for: TRPV4 overactivation enhances cellular contractility and drives ocular hypertension in TGFβ2 overexpressing eyes
Source: bioRxiv. 2025 May 19:2024.11.05.622187. Originally published 2024 Nov 7. Preprint. [Version 2] doi: 10.1101/2024.11.05.622187 (PMC11580928; doi:10.1101/2024.11.05.622187)

bioRxiv preprint doi: https://doi.org/10.1101/2024.11.05.622187; this version posted May 19, 2025. The copyright holder for this preprint (which was not certified by peer review) is the author/funder, who has granted bioRxiv a license to display the preprint in perpetuity. It is made available under aCC-BY 4.0 International license.

Supplemental Information

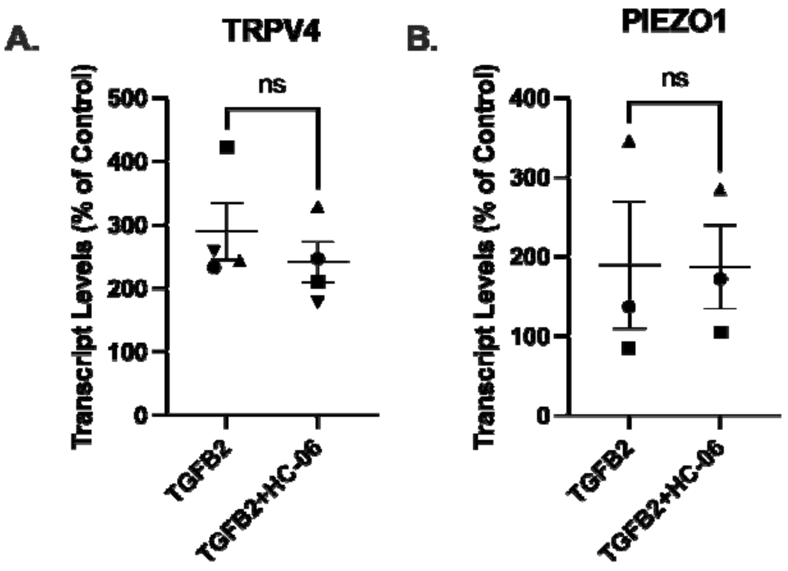

Supplementary Figure S1: No significant difference was seen in *TRPV4* or *PIEZO1* expression between pTM samples treated with TGFβ2 (1 ng/mL) alone or TGFβ2 + TRPV4 antagonist HC-06 (5μM) for five days. N=3-4 independent experiments. Within each gene, symbols indicate paired samples. Wilcoxon matched-pairs signed rank test and paired t-test used respectively.

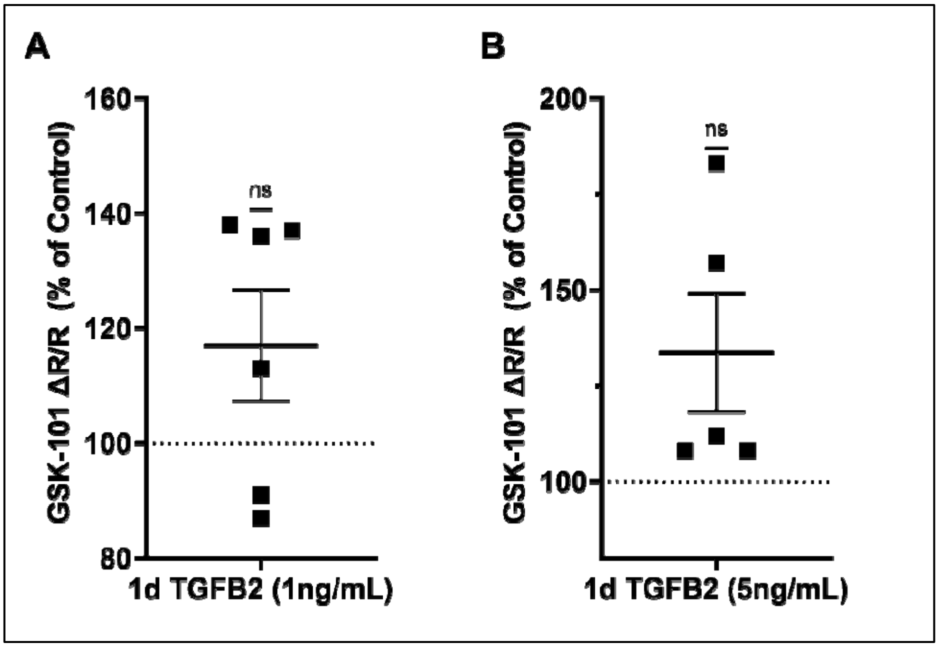

Supplementary Figure S2: TGFβ2 concentrations of 1 ng/mL (**A)** and 5 ng/mL (**B)** did not significantly increase TRPV4-induced calcium influx with respect to control cells. Individual statistical analysis of experiments shown in Fig. 3*A* (1 ng/mL: *P* = 0.138, 5ng/mL: *P* = 0.095), one sample t-test.

bioRxiv preprint doi: https://doi.org/10.1101/2024.11.05.622187; this version posted May 19, 2025. The copyright holder for this preprint (which was not certified by peer review) is the author/funder, who has granted bioRxiv a license to display the preprint in perpetuity. It is made available under aCC-BY 4.0 International license.

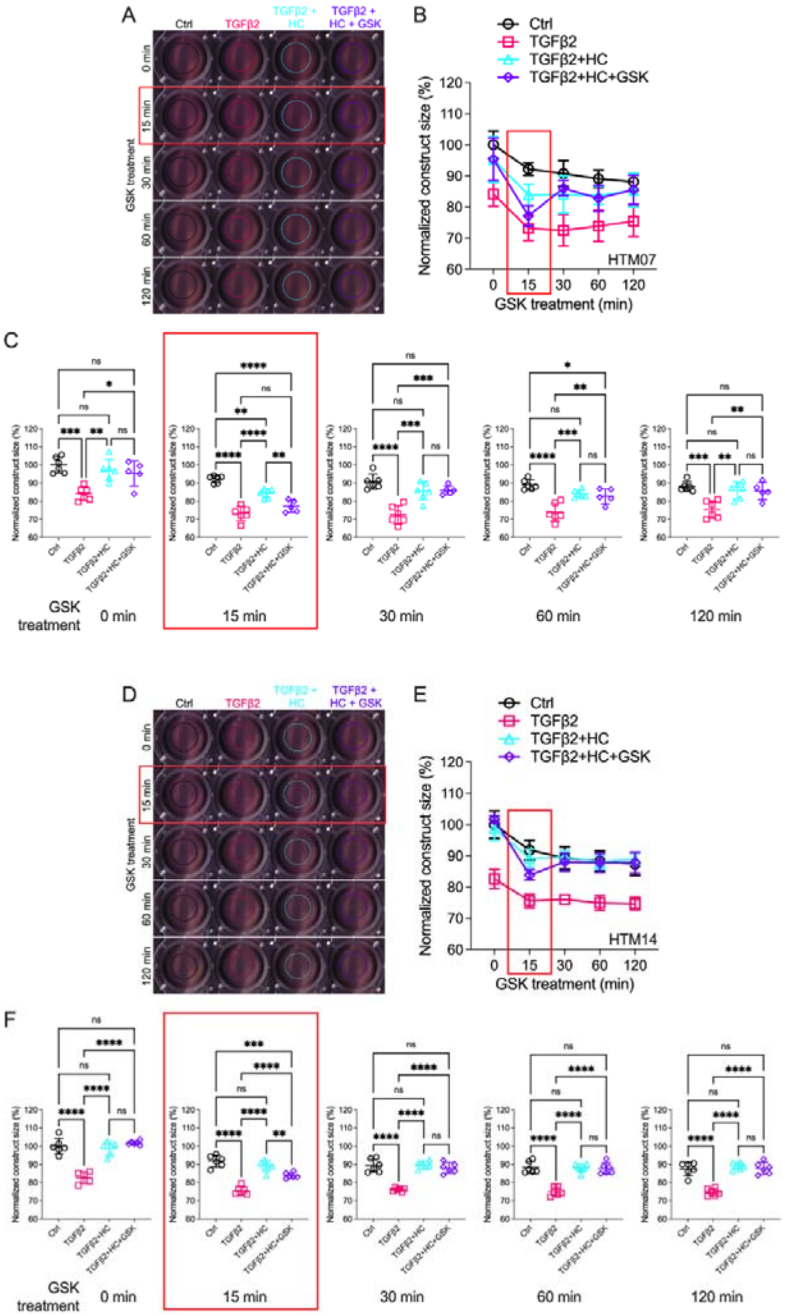

<u>Supplementary Figure S3: TRPV4 activation is obligatory for TGFβ2-induced TM cell contractions</u>. (**A, D**) Representative longitudinal 24-well plate scans of collagen type I hydrogels seeded with two distinct pTM strains (pTM 1: **A**-**C**, pTM 2: **D**-**F**) subjected to the different treatments as in Fig. 4. (**B, E**) Longitudinal quantification of hydrogel construct size. (**C, F**) Detailed comparisons between groups at each experimental time point (N = 6 experimental replicates/ pTM strain). One-way ANOVA with Tukey multiple comparisons test, data in (**B, D**) shows individual data points over mean ± SEM * P < 0.05, ** P < 0.01, *** P < 0.001, **** P < 0.0001.

bioRxiv preprint doi: https://doi.org/10.1101/2024.11.05.622187; this version posted May 19, 2025. The copyright holder for this preprint (which was not certified by peer review) is the author/funder, who has granted bioRxiv a license to display the preprint in perpetuity. It is made available under aCC-BY 4.0 International license.

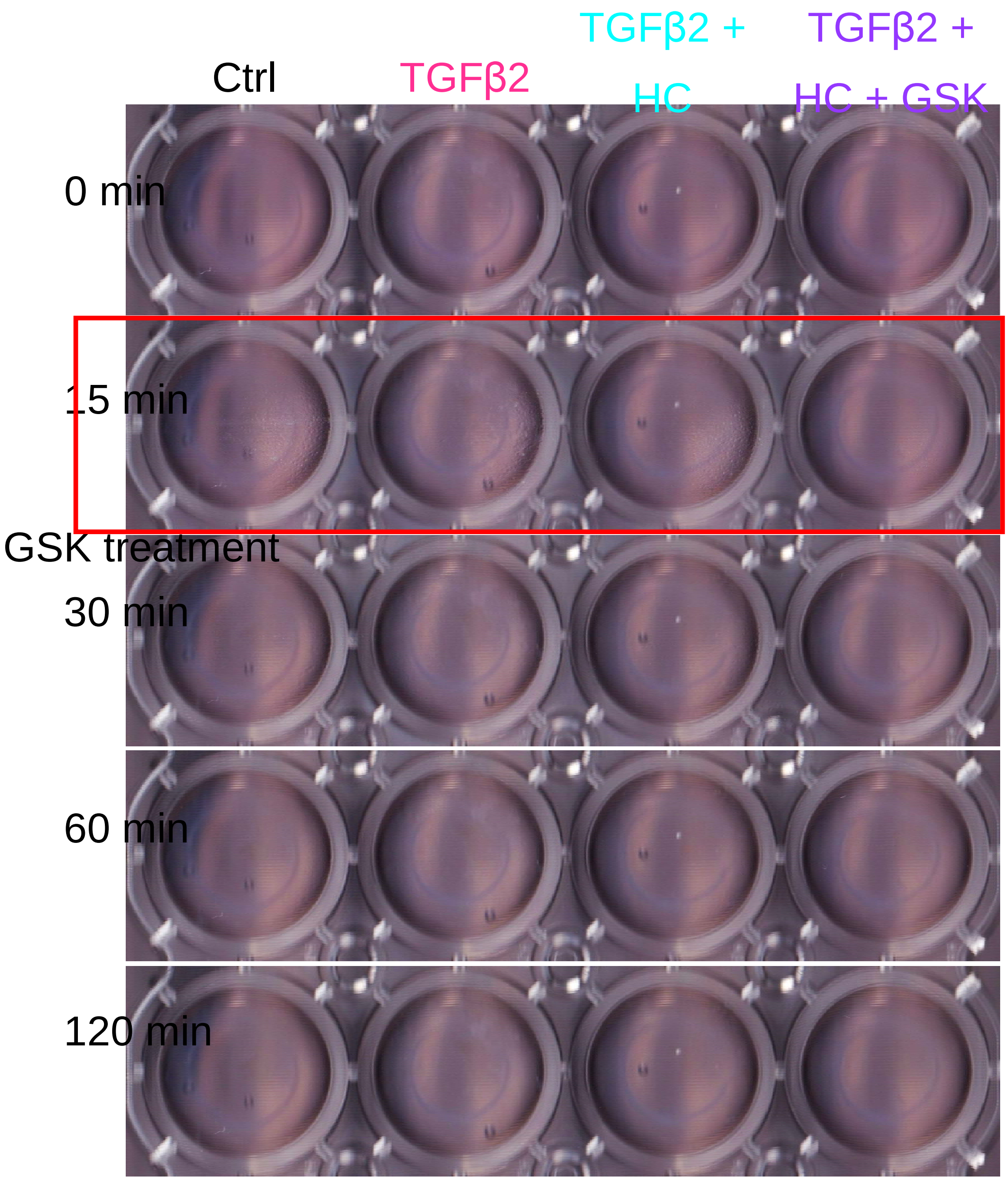

Supplementary Figure S4: Detailed view of pTM seeded collagen constructs used in Figure 4 and S3. High resolution representative image of collagen gels used for contractility experiments without circle around periphery of gel.

bioRxiv preprint doi: https://doi.org/10.1101/2024.11.05.622187; this version posted May 19, 2025. The copyright holder for this preprint (which was not certified by peer review) is the author/funder, who has granted bioRxiv a license to display the preprint in perpetuity. It is made available under aCC-BY 4.0 International license.

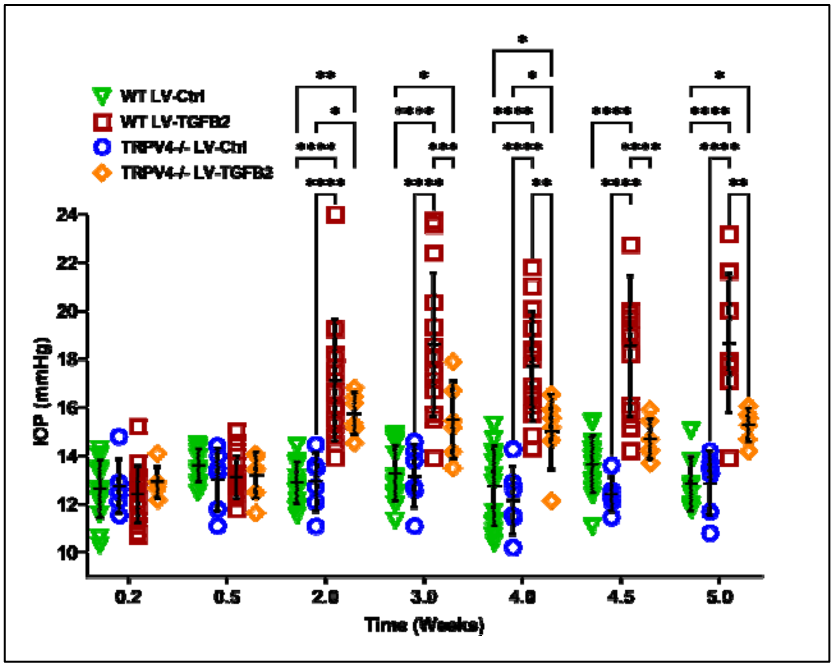

Supplementary Figure S5: Expansion of Fig. 5*D*. IOP in LV-TGFβ2-injected eyes was significantly elevated compared to both LV-Ctrl injected WT and *Trpv4*$^{-/-}$ eyes, as well as LV-TGFβ2-injected *Trpv4*$^{-/-}$ eyes (N = 6 eyes/condition).

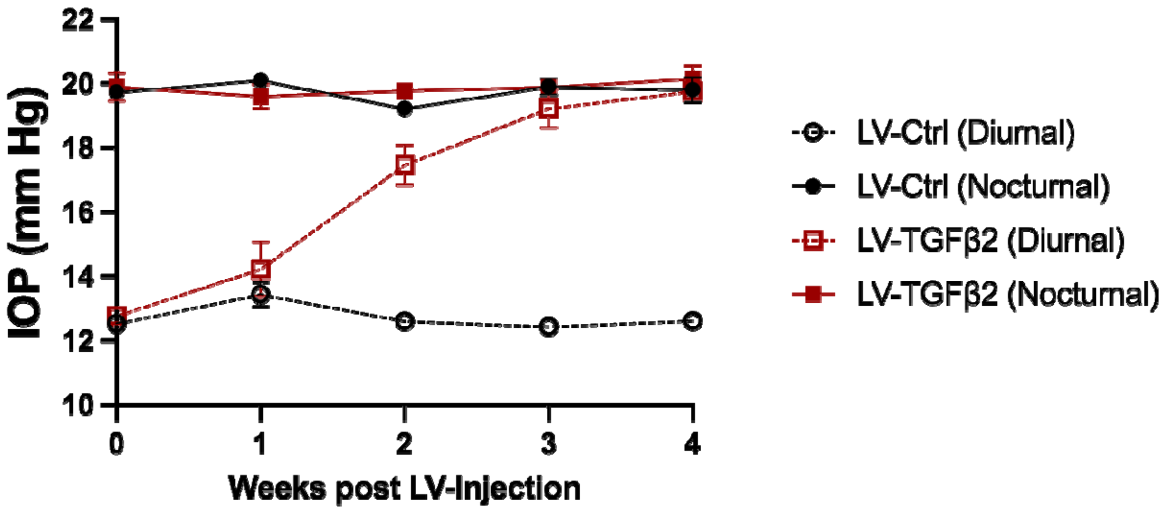

Supplementary Figure S6: Nocturnal IOP is not significantly affected by LV-TGFβ2 overexpression. Expanded time series of IOP measured weekly from a second cohort of mouse eyes (Fig. 6B) injected with LV-Ctrl (n = 6 eyes) or LV-TGFβ2 (n = 4 eyes). LV-TGFβ2 resulted in elevated diurnal IOP which gradually approached the IOP seen in nocturnal measurements, but did not further elevate IOP above nocturnal values. In this cohort, both diurnal and nocturnal measurements were made in awake animals.

bioRxiv preprint doi: https://doi.org/10.1101/2024.11.05.622187; this version posted May 19, 2025. The copyright holder for this preprint (which was not certified by peer review) is the author/funder, who has granted bioRxiv a license to display the preprint in perpetuity. It is made available under aCC-BY 4.0 International license.

Supplementary Information: Uncropped Western blots.

bioRxiv preprint doi: https://doi.org/10.1101/2024.11.05.622187; this version posted May 19, 2025. The copyright holder for this preprint (which was not certified by peer review) is the author/funder, who has granted bioRxiv a license to display the preprint in perpetuity. It is made available under aCC-BY 4.0 International license.

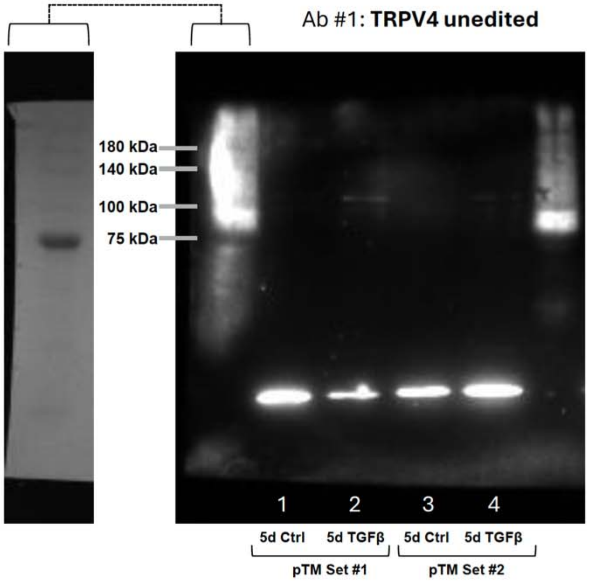

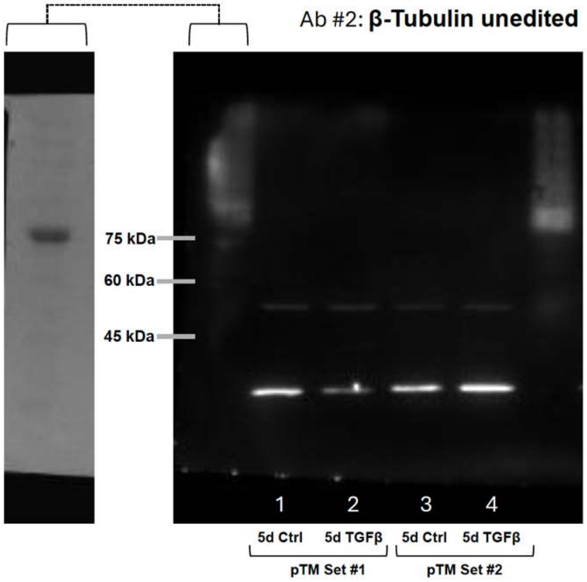

Ab #2: **β-Tubulin uncropped**

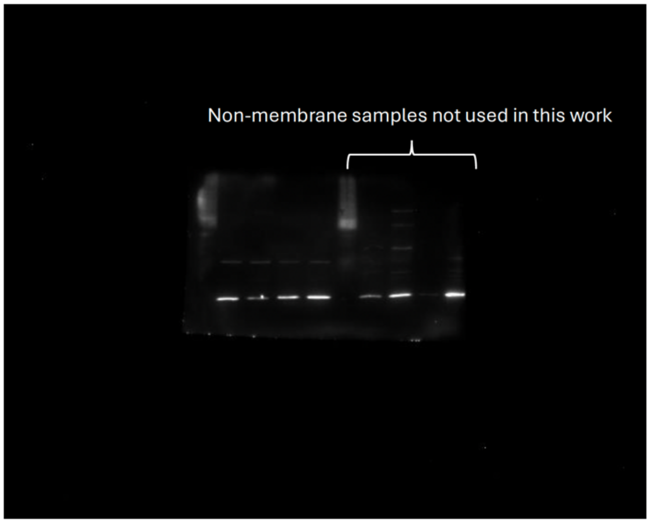

Ab #1: **TRPV4, uncropped**

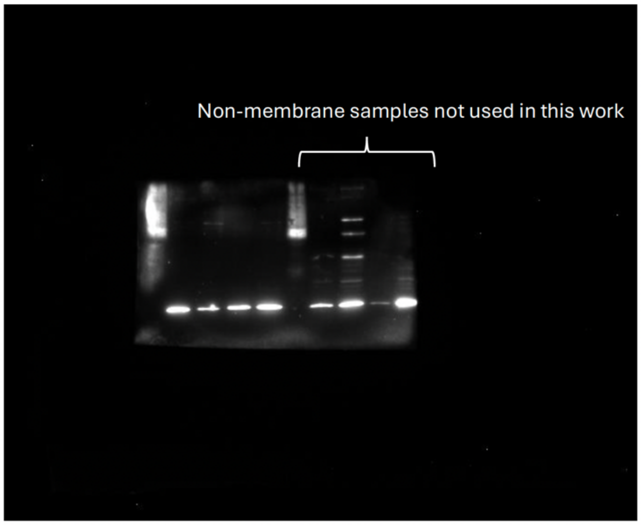

Supplement: 1 [file NIHPP2024.11.05.622187v2-supplement-1.pdf]
